# Antihyperglykämische Therapie bei Diabetes mellitus Typ 2 (Update 2023)

**DOI:** 10.1007/s00508-023-02186-4

**Published:** 2023-04-20

**Authors:** Martin Clodi, Heidemarie Abrahamian, Helmut Brath, Guntram Schernthaner, Johann Brix, Bernhard Ludvik, Heinz Drexel, Christoph H. Saely, Peter Fasching, Gersina Rega-Kaun, Bernhard Föger, Claudia Francesconi, Elke Fröhlich-Reiterer, Alexandra Kautzky-Willer, Jürgen Harreiter, Anton Luger, Michael Resl, Michaela Riedl, Yvonne Winhofer, Sabine E. Hofer, Friedrich Hoppichler, Joakim Huber, Susanne Kaser, Claudia Ress, Monika Lechleitner, Felix Aberer, Julia K. Mader, Harald Sourij, Hermann Toplak, Bernhard Paulweber, Lars Stechemesser, Thomas Pieber, Rudolf Prager, Harald Stingl, Thomas Stulnig, Birgit Rami-Merhar, Heinz Drexel, Michael Roden, Christian Schelkshorn, Thomas C. Wascher, Raimund Weitgasser, Sandra Zlamal-Fortunat

**Affiliations:** 1 ICMR – Institute for Cardiovascular and Metabolic Research, Johannes Kepler Universität Linz JKU Linz, Altenberger Straße 69, 4040 Linz, Österreich; 2 Abteilung für Innere Medizin mit Diabetologie, Gastroenterologie und Hepatologie, Rheumatologie und Intensivmedizin, , Konventhospital der Barmherzigen Brüder Linz, Linz, Österreich; 3 Privates Institut für Medizin & NLP, Wissenschaftliches Institut gemäß BundesstatistikG 2008 ÖNACE-CODE: 72.19-0, Wien, Österreich; 4 Sigmund Freud Privatuniversität Medizin, Campus Prater, Wien, Österreich; 5 Diabetes- und Fettstoffwechselambulanz, Mein Gesundheitszentrum Favoriten, Wien, Österreich; 6 Universität Wien, Wien, Österreich; 7 1. Medizinische Abteilung mit Diabetologie, Endokrinologie und Nephrologie, Krankenanstalt Rudolfstiftung, Wien, Österreich; 8 Vorarlberg Institute for Vascular Investigation and Treatment (VIVIT), Feldkirch, Österreich; 9 Abteilung für Innere Medizin I, Akademisches Lehrkrankenhaus Feldkirch, Feldkirch, Österreich; 10 5. Medizinische Abteilung für Endokrinologie, Rheumatologie und Akutgeriatrie, Wilhelminenspital der Stadt Wien, Wien, Österreich; 11 Abteilung für Allgemein Innere Medizin, Rottal Inn Kliniken, Pfarrkirchen, Deutschland; 12 Sonderkrankenanstalt Rehabilitationszentrum Alland, Alland, Österreich; 13 Universitätsklinik für Kinder- und Jugendheilkunde, Medizinische Universität Graz, Graz, Österreich; 14 Klinische Abteilung für Endokrinologie und Stoffwechsel, Universitätsklinik für Innere Medizin III, Medizinische Universität Wien, Wien, Österreich; 15 Gender Medicine Unit, Klinische Abteilung für Endokrinologie und Stoffwechsel, Universitätsklinik für Innere Medizin III, Medizinische Universität Wien, Wien, Österreich; 16 Department für Pädiatrie 1, Medizinische Universität Innsbruck, Innsbruck, Österreich; 17 Abteilung für Innere Medizin, Krankenhaus der Barmherzigen Brüder Salzburg, Salzburg, Österreich; 18 Interne Abteilung mit Akutgeriatrie und Palliativmedizin, Franziskus Spital, Standort Landstraße, Wien, Österreich; 19 Department für Innere Medizin I, Medizinische Universität Innsbruck, Innsbruck, Österreich; 20 Christian Doppler Labor für Insulinresistenz, Medizinische Universität Innsbruck, Innsbruck, Österreich; 21 Avomed – Arbeitskreis für Vorsorgemedizin zbd Gesundheitsförderung in Tirol, Innsbruck, Österreich; 22 Klinische Abteilung für Endokrinologie und Diabetologie, Universitätsklinik für Innere Medizin, Medizinische Universität Graz, Graz, Österreich; 23 Universitätsklinik für Innere Medizin I, mit Gastroenterologie, Hepatologie, Nephrologie, Stoffwechsel und Diabetologie, Paracelsus Medizinische Privatuniversität, Salzburg, Österreich; 24 Universitätsklinik für Innere Medizin, Medizinische Universität Graz, Graz, Österreich; 25 3. Medizinische Abteilung, Karl Landsteiner Institut für Stoffwechselerkrankungen und Nephrologie, Krankenhaus Hietzing der Stadt Wien, Wien, Österreich; 26 Karl-Landsteiner-Universität für Gesundheitswissenschaften, Krems, Österreich; 27 Abteilung für Innere Medizin, Landeskliniken Baden–Mödling, Standort Baden, Baden, Österreich; 28 3. Medizinische Abteilung mit Stoffwechselerkrankungen und Nephrologie, Krankenhaus Hietzing Wien, Wien, Österreich; 29 Universitätsklinik für Kinder- und Jugendheilkunde, Medizinische Universität Wien, Wien, Österreich; 30 Chair der ESC-Working Group „Cardiovascular Pharmacotherapy“, Sophia Antipolis, Frankreich; 31 Drexel University College of Medicine, Philadelphia, PA USA; 32 ESC-Working Group „Cardiovascular Pharmacotherapy“, Sophia Antipolis, Frankreich; 33 Drexel University College of Medicine, Philadelphia, PA USA; 34 Private Universität im Fürstentum Liechtenstein, Triesen, Liechtenstein; 35 Klinik für Endokrinologie und Diabetologie, Medizinische Fakultät, Heinrich-Heine-Universität, Düsseldorf, Deutschland; 36 Deutsches Zentrum für Diabetesforschung, DZD e. V., München-Neuherberg, Deutschland; 37 Institut für Klinische Diabetologie, Deutsches Diabetes-Zentrum (DDZ), Leibniz-Zentrum für Diabetesforschung, Düsseldorf, Deutschland; 38 1. Medizinische Abteilung, Landesklinikum Stockerau, Stockerau, Österreich; 39 1. Medizinische Abteilung, Mein Hanusch-Krankenhaus, Wien, Österreich; 40 Abteilung für Innere Medizin, Privatklinik Wehrle-Diakonissen, Salzburg, Österreich; 41 Universitätsklinik für Innere Medizin I, LKH Salzburg – Universitätsklinikum der Paracelsus Medizinischen Privatuniversität, Salzburg, Österreich; 42 Abteilung für Innere Medizin und Gastroenterologie, Klinikum Klagenfurt am Wörthersee, Klagenfurt, Österreich

**Keywords:** Diabetes mellitus Typ 2, Therapie Blutzuckersenkung, Diabetes mellitus Type 2, Therapy Glucose lowering

## Abstract

Die Hyperglykämie ist wesentlich an der Entstehung der Spätkomplikationen bei an Diabetes mellitus Typ 2 erkrankten Patienten/Patientinnen beteiligt. Während Lebensstilmaßnahmen die Eckpfeiler jeder Diabetestherapie bleiben, benötigen im Verlauf die meisten Patienten/Patientinnen mit Typ 2 Diabetes eine medikamentöse Therapie. Bei der Definition individueller Behandlungsziele stellen die Therapiesicherheit, die Effektivität sowie substanzspezifische, kardiovaskuläre Effekte der Therapie die wichtigsten Faktoren dar. In dieser Leitlinie haben wir die rezenten evidenzbasierten Daten für die klinische Praxis zusammengestellt.

## Abstract (en)

Hyperglycemia significantly contributes to complications in patients with diabetes mellitus. While lifestyle interventions remain cornerstones of disease prevention and treatment, most patients with type 2 diabetes will eventually require pharmacotherapy for glycemic control. The definition of individual targets regarding optimal therapeutic efficacy and safety as well as cardiovascular effects is of great importance. In this guideline we present the most current evidence-based best clinical practice data for healthcare professionals.

## Einleitung

Die Hyperglykämie der an Diabetes mellitus Typ 2 erkrankten Patient:innen trägt entscheidend zur Pathogenese mikrovaskulärer Komplikationen bei, ist Cofaktor bei der Entwicklung makrovaskulärer Erkrankungen und ist ursächlich für direkte zelluläre Schädigungen.

Das primäre Ziel einer antihyperglykämischen Therapie ist daher, neben dem Vermeiden von akuten Komplikationen der Hyperglykämie, die Prävention zellulärer und vaskulärer Komplikationen. Des Weiteren stellen Symptomfreiheit, der Erhalt der Lebensqualität, sowie Komaprophylaxe wesentliche Therapieziele dar.

Der Zusammenhang zwischen Hyperglykämie und Mikroangiopathie ist im Vergleich zur Makroangiopathie stärker ausgeprägt und linear. Damit ist eine Prävention durch verbesserte Blutzuckereinstellung im Bereich der Mikroangiopathie leichter zu erreichen.

## Therapieziele

Zu den allgemeinen Zielen der Therapie zählen

- Vermeiden von Akutkomplikationen,
- Vermeiden von Folgekomplikationen,
- Symptomfreiheit sowie Erhalt bzw. Wiederherstellung der Lebensqualität.

Bei *Prädiabetes* wird basierend auf der aktuell verfügbaren Datenlage die Umsetzung lebensstilmodifizierender Maßnahmen mit dem Ziel einer Gewichtsreduktion und des Muskelaufbaus empfohlen. Eine medikamentöse Therapie kann in Erwägung gezogen werden (z. B. Metformin).

Für die antihyperglykämische Therapie gelten unten angeführte Zielwerte. Als Mittel der ersten Wahl bei Patient:innen ohne Komorbidität sollte Metformin eingesetzt werden. Bei Patient:innen mit kardiorenalen Komorbiditäten sind neben Metformin SGLT-2-Hemmer und GLP-1-Analoga die Mittel der ersten Wahl.

Bei einer Kontraindikation oder einer Unverträglichkeit gegenüber Metformin muss je nach individuellen Erfordernissen des Patienten ein anderes der verfügbaren Präparate angewandt werden.

**Figure 1:**
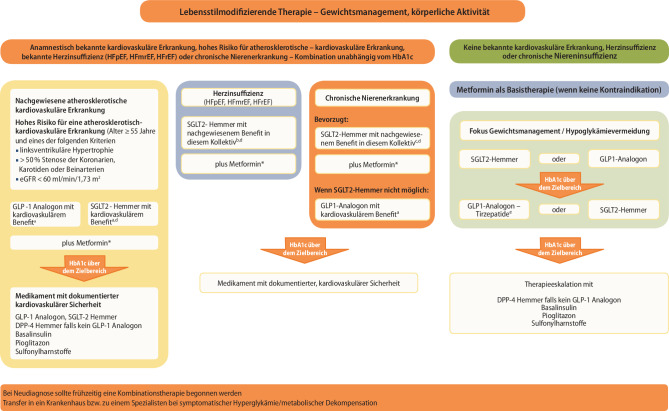

Sollte bei einer Monotherapie mit einem Medikament der Zielwert nicht erreicht werden, muss eine Therapiemodifikation durchgeführt werden. Möglichkeiten hierfür sind in der Abb. 1 zum Teil in Analogie zu den aktuell gültigen Leitlinien der Europäischen bzw. Amerikanischen Diabetesgesellschaft dargestellt. Weiterhin existieren nur wenige Daten mit längeren Nachbeobachtungszeiträumen zur Auswahl der Medikamente bei Mehrfachkombinationen.

Große, randomisiert kontrollierte Studien konnten substanzspezifische kardiovaskuläre Effekte bei SGLT-2-Hemmern (Empagliflozin, Canagliflozin, Dapagliflozin) wie auch bei GLP-1-Analoga (Liraglutid, Dulaglutid und Semaglutid) dokumentieren.

Basierend auf den Daten der Gliflozine bezüglich Herzinsuffizienz (erhaltene HFpEF oder reduzierte Linksventrikelfunktion HFrEF) und chronische Niereninsuffizienz müssen diese Diagnosen bezüglich der weiteren Therapieentscheidung unabhängig vom HbA_1c_ zusätzlich berücksichtigt werden.

Wird unter Metformin der individuell festgelegte HbA_1c_-Zielwert nicht erreicht, so wird bei der erforderlichen Therapieeskalation die Berücksichtigung kardiovaskulärer Komorbiditäten empfohlen.

Das HbA_1c_ stellt die primäre Richtgröße der Stoffwechselkontrolle dar. Postprandiale Glukose und Nüchternglukose stellen weitere Richtgrößen dar.

### FACT-Box

- Basis jeder Diabetestherapie ist eine lebenslange Lebensstilmodifikation (Gewichtsreduktion/Bewegung).
- Bei kurzer Diabetesdauer und langer Lebenserwartung wird ein HbA_1c_-Zielwert ≤ 6,5 % empfohlen, sofern das ohne relevante Nebenwirkungen der Therapie erreicht werden kann.
- Ein HbA_1c_-Ziel < 7,0 % ist für einen ausreichenden mikro- und makrovaskulären Schutz notwendig.
- Metformin, SGLT-2-Hemmer und GLP-1-Agonisten nehmen eine zentrale Rolle in der Behandlung ein.
- Derzeit gibt es für Empagliflozin, Canagliflozin, Dapagliflozin, Liraglutid, Dulaglutid, Semaglutid und Pioglitazon (sekundärer Endpunkt) positive, substanzspezifische kardiovaskuläre Daten aus placebokontrollierten, randomisierten prospektiven Studien (RCT).
- Bei Herzinsuffizienz (mit erhaltener HFpEF oder reduzierter Linksventrikelfunktion HFrEF) sollte unabhängig vom HbA_1c_ ein Gliflozin (Dapagliflozin oder Empagliflozin) verabreicht werden.
- Bei chronischer Niereninsuffizienz sollte ebenfalls unabhängig vom HbA_1c_ ein Gliflozin zusätzlich etabliert werden.

Generell ist für die meisten Patient:innen ein HbA_1c_ < 7,0 % für einen ausreichenden mikrovaskulären, makrovaskulären und zellulären Schutz notwendig.

Bei Patient:innen mit kurzer Diabetesdauer, langer Lebenserwartung und keiner relevanten kardiovaskulären Komorbidität ist ein HbA_1c_-Wert unter 6,5 % sinnvoll.

Kann dieses Therapieziel nicht komplikationslos und ohne große Gefahr für Hypoglykämien erreicht werden, so ist auch ein HbA_1c_-Zielwert ≤ 7,0 % ausreichend. Die Reduktion von mikrovaskulären sowie auch makrovaskulären Spätkomplikationen wurde mittlerweile nachgewiesen.

Bei Patient:innen mit mehreren, schweren Hypoglykämien und/oder eingeschränkter Lebenserwartung sowie multiplen Spätkomplikationen kann ein HbA_1c_-Zielwert bis zu 8,0 % als ausreichend erachtet werden.

Neben dem HbA_1c_ stellen die Nüchtern- und die postprandiale Glukose sekundäre Richtgrößen dar. Dementsprechend sollte die Nüchternglukose unter 130 mg/dl (ideal < 110 mg/dl) liegen bzw. die postprandiale Glukose (2 h nach einer Mahlzeit) maximal 180 mg/dl betragen.

Im Vergleich zur Version aus dem Jahr 2019 empfehlen wir aufgrund der neuen Studiendaten in Anlehnung an den EASD/ADA Konsensus neben der etablierten kardiovaskulären Erkrankung auch das hohe Risiko für eine atherosklerotische kardiovaskuläre Erkrankung als Indikation für eine Therapie mit GLP1-Rezeptoragonisten oder SGLT-2-Hemmer mit nachgewiesenem kardiovaskulären Benefit (Tab. 1) anzusehen. Diese Therapie sollte unabhängig vom HbA_1c_ initiiert werden.

**Table 1:** 

Klasse	HbA_1c_	Hypoglykämie	Vorteile	Nachteile
Metformin	1–2 %	Nein	Gewichtsneutralität, Reduktion makrovaskulärer Ereignisse	KI und GI Nebenwirkungen
SGLT-2-Hemmer	0,5–1 %	Nein	Empagliflozin, Canagliflozin und Dapagliflozin reduzieren kardiovaskuläre Ereignisse, positive Daten bei HFpEF und HFrEF, Gewichtsreduktion	Genitale Infekte, sehr selten Auslöser normoglykämischer Ketoazidosen, Hinweise auf erhöhtes Amputationsrisiko (für Canagliflozin)
GLP-1-Rezeptor-Agonisten	1–2 %	Nein	Gewichtsreduktion, Reduktion kardiovaskulärer Ereignisse unter Liraglutid, Dulaglutid und Semaglutid	Nausea, subkutane Injektion
GLP-1-GIP-Agonisten	2–2,3 %	Nein	Ausgeprägte Gewichtsreduktion	Nausea, subkutane Injektion
Pioglitazon	1–2 %	Nein	Reduktion kardiovaskulärer Ereignisse	Gewichtszunahme, periphere Ödeme, Frakturen bei Frauen
DPP-4-Hemmer	0,5 –1 %	Nein	Gewichtsneutral	Moderate Wirksamkeit
Sulfonylharnstoffe	1–2 %	Ja	Rasche Blutzuckersenkung	Mögliche Gewichtszunahme, Hypoglykämien
Glinide	1–2 %	Ja	Verbesserte postprandiale BZ Kontrolle	Dreimal tägliche Dosierung, mögliche Gewichtszunahme
Alpha-Glucosidase-Inhibitoren	−1,0 %	Nein	Verbesserte postprandiale BZ-Kontrolle, gewichtsneutral	GI Nebenwirkungen
Insulin	2 %	Ja	Keine Dosisobergrenze, viele Arten, flexible Regelungen	Gewichtszunahme, Hypoglykämie

(Alter ≥ 55 Jahre und eines der folgenden Kriterien):

- linksventrikuläre Hypertrophie
- > 50 % Stenose der Koronarien, Carotiden oder Beinarterien
- eGFR < 60 ml/min/1,73 m^2^
- Albuminurie

## Orale Antidiabetika

### Metformin

Metformin wirkt primär durch eine Hemmung der Glukoneogenese mit Senkung der (Nüchtern)-Glukoseproduktion, nachfolgend tritt eine Verbesserung der hepatischen und peripheren Insulinsensitivität ein. In der Monotherapie wird durch Metformin eine HbA_1c_-Reduktion von ca. 1,5 % bei einer Senkung des Nüchternblutzuckers um 30–40 mg/dl erreicht. Die Metformintherapie wird mit zweimal 500–850 mg pro Tag begonnen und sollte langsam (1–2 wöchentlich) bis zu 2000 mg um jeweils 500 mg/Woche gesteigert werden. Generell ist auch bei übergewichtigen, geriatrischen Patient:innen eine initiale Therapie mit Metformin zu empfehlen. Der appetithemmende und damit gewichtsreduzierende Effekt von Metformin kann aber gerade beim geriatrischen Patienten aufgrund der Gefahr einer Malnutrition unerwünscht sein (siehe Geriatrieleitlinie). Gastrointestinale Nebenwirkungen werden bei dieser schrittweisen Steigerung der Tagesdosis reduziert. Als Kontraindikationen für die Metformintherapie gelten eine schwere Einschränkung der Nierenfunktion, dekompensierte Lebererkrankungen, akute Pankreatitis, Alkoholismus, Malnutrition, eine dekompensierte Herzinsuffizienz und/oder andere hypoxische Situationen. Metformin darf bei Patient:innen mit eGFR-Werten zwischen 30–45 ml/min/1,73 m^2^ bei Fehlen von anderen Risikofaktoren für Laktatazidosen in einer Dosierung von 1000 mg, täglich aufgeteilt auf zwei Dosen, angewandt werden. Die glomeruläre Filtrationsrate sollte mit Hilfe einer entsprechenden Formel (z. B. CKD-EPI oder MDRD) evaluiert, zumindest alle 3–6 Monate kontrolliert werden. Falls die eGFR unter 30 ml/min/1,73 m^2^ abfällt, muss Metformin abgesetzt werden. Bei interkurrierenden schweren Erkrankungen (schwere Infektionen) sowie auch bei Diarrhoe und Exsikkose und der Applikation von Kontrastmittel sollte Metformin ebenso vorübergehend pausiert werden. Da es unter einer Therapie mit Metformin zu einem Vitamin B12-Mangel kommen kann, wird empfohlen, die Vitamin B12-Spiegel jährlich im Rahmen der regelmäßigen Blutabnahmen zu kontrollieren.

### SGLT2-Inhibitoren

Der Natrium-Glucose-Cotransporter (SGLT2) ist verantwortlich für den größten Teil der Glukose-Resorption im proximalen Tubulus der Niere. Die SGLT-2-Inhibitoren bewirken daher eine kontrollierte Glukosurie und damit eine Reduktion der Hyperglykämie. Die Wirkung der SGLT-2-Hemmer ist unabhängig von Insulin. Die in Österreich aktuell verfügbaren Substanzen sind Dapagliflozin, Empagliflozin und Canagliflozin. Prinzipiell können SGLT-2-Hemmer in jeder Kombination eingesetzt werden. Neben der Blutzuckersenkung (das HbA_1c_ sinkt um 0,5–1 %) kommt es zu einer Senkung des Blutdruckes (2–4/1–2 mm Hg) und zu einer Gewichtsabnahme (−2 bis −3 kg), wobei das LDL-Cholesterin gering ansteigt (etwa 5 %). Aufgrund der glukosurischen Wirkung dieser Medikamentenklasse ergibt sich auch ein diuretischer Effekt. Das diskret erhöhte Risiko für Beinamputationen in der CANVAS-Studie, wurde in der CREDENCE-Studie nicht bestätigt.

Empagliflozin, Dapagliflozin und Canagliflozin bewirken eine signifikante Reduktion sowohl kardiovaskulärer als auch renaler Endpunkte. Darüber hinaus konnte sowohl für Dapagliflozin als auch für Empagliflozin bei Patient:innen mit Herzinsuffizienz (erhaltene HFpEF – oder reduzierte Linksventrikelfunktion HFrEF) eine signifikante Reduktion der Hospitalisationen aufgrund von Herzinsuffizienz registriert werden.

Daten aus der DAPA–CKD Studie (Dapagliflozin), der EMPA-Kidney als auch aus der CREDENCE-Studie (Canagliflozin) unterstützen die Empfehlung, dass bei chronischer Niereninsuffizienz, SGLT-2-Hemmer mit Evidenz für Reduktion der Progression der chronischen Niereninsuffizienz eingesetzt werden sollen. Empagliflozin und Canagliflozin müssen erst ab einer eGFR < 30 ml/min/1,73 m^2^ und Dapagliflozin ab einer eGFR < 25 ml/min/1,73 m^2^ abgesetzt werden.

Unter Therapie mit Gliflozinen wurde das Auftreten von euglykämischen Ketoazidosen vereinzelt berichtet. Folgende, mögliche Risikofaktoren sind bisweilen bekannt: Infektionen, „Low-Carbohydrate Diet“, Reduktion/Absetzen einer laufenden Insulintherapie, Absetzen von oralen Insulinsekretagoga, Diabetes mellitus Typ 1 und Alkoholmissbrauch. Entsprechend einer Warnung der EMA sollten zur Risikominimierung des Auftretens einer seltenen, aber möglichen Ketoazidose unter den Gliflozinen generell einige Vorsichtsmaßnahmen eingehalten werden. Bei Verordnung des Medikamentes müssen die Patient:innen über die Symptome der Ketoazidose (Polyurie, Polydipsie, Lethargie, unklare Gewichtsabnahme, Bauchschmerzen) aufgeklärt werden. Sollte sich eine Ketoazidose bestätigen, müssen die SGLT-2-Hemmer sofort abgesetzt und eine stationäre Therapie eingeleitet werden. Bei Patienten mit Risikofaktoren für eine Ketoazidose (geringe Insulinsekretionsreserve, Erkrankungen, welche die Nahrungsmitteleinnahme reduzieren, schwere Dehydratation, plötzliche Insulinreduktionen, Operationen oder Alkoholabusus) sollten die Gliflozine vorsichtig eingesetzt werden. Generell sollen Gliflozine bei Operationen und/oder schweren Erkrankungen pausiert werden.

Der aktuell in Österreich nicht verfügbare duale SGLT-1- und SGLT-2-Hemmer Sotagliflozin konnte in der SOLOIST-WHF-Studie und der SCORED-Studie eine signifikante Reduktion des Endpunktes (Kardiovaskulärer Tod, Hospitalisation oder akuter ambulanter Besuch wegen Herzinsuffizienz) bewirken [1, 2].

### GLP-1-Rezeptor-Agonisten

Glucagon-like Peptide‑1 (GLP-1) Rezeptor-Agonisten (Exenatid, Liraglutid, Semaglutid, Lixisenatid, Dulaglutid) führen zu einer Glukose-abhängigen Steigerung der pankreatischen Insulinsekretion, Hemmung der Glucagonfreisetzung und der Magenentleerung sowie Auslösung eines Sättigungsgefühls durch Stimulation des Sättigungszentrums im Gehirn. Sie müssen subkutan in Intervallen von einmal täglich bis einmal wöchentlich je nach Substanz verabreicht werden. Neben effektiver Blutzuckerreduktion sind die fehlende Hypoglykämieneigung und eine, in Studien beobachtete Gewichtsreduktion festzuhalten. Gastrointestinale Nebenwirkungen (Übelkeit, Erbrechen) treten häufiger auf als unter Placebo. Im Rahmen der LEADER-Studie bewirkte Liraglutid eine signifikante Senkung des präspezifizierten kardiovaskulären Endpunktes, wobei dies maßgeblich auf einer signifikanten Reduktion kardiovaskulärer Todesfälle basiert. Auch Semaglutid konnte in der SUSTAIN-6-Studie eine signifikante Reduktion des kombinierten primären Endpunktes (Kardiovaskulärer Tod, nicht-tödlicher Myokardinfarkt, nicht tödlicher Insult) erreichen. In der REWIND-Studie konnte der primäre Endpunkt, ein kombinierter Endpunkt aus kardiovaskulärem Tod, nicht-tödlichem Myokardinfarkt und nicht-tödlichem Schlaganfall (3-Punkt MACE) mittels Dulaglutid um relative 12 %, signifikant reduziert werden (HR 0,88, 95 %-Konfidenzintervall 0,79–0,99).

In der ELIXA-Studie (Lixisenatid) und in der EXCSEL-Studie (Exenatid 1 × wöchentlich) wurde die kardiovaskuläre Sicherheit für diese Substanzen belegt, allerdings konnte kein substanzspezifischer kardiovaskulärer Zusatznutzen gezeigt werden.

### Duale Agonisten GLP-1/GIP

In rezent publizierten Studien konnte der Vorteil einer Therapieerweitung mit dem (in Österreich derzeit noch nicht verfügbaren) dualen Wirkstoff Tirzepatide, einer Kombination eines Glucose-dependent insulinotropic Peptides (GIP) mit einem GLP1-RA, gegenüber eine Therpieerweiterung mit Insulin Degludec aufgezeigt werden. Bei Patienten mit Typ 2 Diabetes und Metformin-Vortherapie fand sich unter Tirzepatide eine stärkere Reduktion des HbA_1c_-Wertes, eine Reduktion des Körpergewichts und ein niedrigeres Hypoglykämierisiko [3]. Tirzepatide ist von der EMA bereits zugelassen worden, zum aktuellen Zeitpunkt in Österreich aber noch nicht verfügbar.

### Gliptine

Dipeptidyl-Peptidase-IV-Inhibitoren (Sitagliptin, Vildagliptin, Saxagliptin, Linagliptin, Alogliptin) als Abbauhemmer des körpereigenen GLP‑1 führen zu einer Glukose-abhängigen Steigerung der pankreatischen Insulinsekretion und Hemmung der Glucagonfreisetzung. Diese Substanzen zeigen keine Hypoglykämieneigung und sind gewichtsneutral. Sie werden in der Monotherapie als auch in Kombination mit Metformin (primär), anderen OADs oder aber in Tripelkombination eingesetzt. In Kombination mit Metformin wird eine substanzeigene HbA_1c_-Senkung von ca. 0,8 % beobachtet.

Rezente Endpunktstudien belegen die kardiovaskuläre Sicherheit von Sita‑, Saxa‑, Lina- und Alogliptin. Es konnte aber kein kardiovaskulärer Zusatznutzen nachgewiesen werden. Im Rahmen der SAVOR-TIMI-53-Studie wurde unter einer Therapie mit Saxagliptin eine signifikant häufigere Rate von Hospitalisierungen aufgrund von Herzinsuffizienz beobachtet. Dieses Resultat bestätigte sich jedoch in den Studien für Sita‑, Lina- und Alogliptin nicht. In den RCTs fand sich eine numerisch erhöhte Inzidenz für Pankreatitis im Promillebereich, diese erreichten jedoch in den Einzelstudien kein Signifikanzniveau.

### Pioglitazon

Pioglitazon erhöht die Insulinsensitivität als Ligand der nukleären Hormonrezeptorfamilie PPAR‑ϒ, über die Regulation der Expression verschiedener insulinempfindlicher Gene. Im Fettgewebe erfolgt eine verstärkte Differenzierung von Präadipozyten zu Adipozyten und damit eine Beeinflussung der metabolischen und endokrinen Aktivität. Die Insulinsensitivität in Leber, Skelettmuskel und im Fettgewebe nimmt zu. In Abhängigkeit vom Ausgangs-HbA_1c_-Wert und der Dosierung reduzieren Glitazone den HbA_1c_-Wert um etwa 1,5 %. Zu den Nebenwirkungen der Glitazontherapie zählen Gewichtszunahme und verstärkte Ödemneigung auf Basis von Flüssigkeitsretention. Kontraindikationen für die Glitazontherapie sind Herzinsuffizienz (wegen Flüssigkeitsretention durch erhöhte Natrium-Rückresorption) und Leberfunktionsstörungen. Pioglitazon selbst hat keine direkten negativen, kardialen Effekte. Bei postmenopausalen Frauen wurde eine Steigerung traumatischer Knochenbrüche beobachtet. In der PROACTIVE-Studie hat Pioglitazon den sekundären Endpunkt (MACE) signifikant gesenkt. Pioglitazon hat auch signifikante Effekte auf die Reduktion von cerebralen Ereignissen bei Menschen mit Prädiabetes gezeigt (IRIS Studie).

### Alpha-Glucosidase-Inhibitoren

Diese Substanzklasse (aktuell in Österreich nur über die internationale Apotheke verfügbar), deren wichtigster Vertreter Acarbose ist, bewirkt über eine Hemmung der intestinalen Kohlenhydratverdauung eine Reduktion vor allem der postprandialen Blutzuckerwerte um 50–60 mg/dl und des HbA_1c_-Werts um 0,7 %. Acarbose kann sowohl als Monotherapie als auch als Therapieergänzung eingesetzt werden. Nebenwirkungen dieser Therapie (Blähungen und Bauchschmerzen) können durch eine schrittweise Dosissteigerung und individuelle Anpassung verringert werden. Im Rahmen der ACE-Studie konnte kein direkter, substanzspezifischer, kardiovaskulärer Zusatznutzen nachgewiesen werden [4].

### Sulfonylharnstoffe

Sulfonylharnstoffe (Gliclazid, Glimepirid, Gliquidon) stimulieren die pankreatische Insulinsekretion und resultieren in einer mittleren zu erwartenden HbA_1c_-Reduktion um 1,5 %. Zu den klinisch relevanten Nebenwirkungen zählt das erhöhte Hypoglykämierisiko. Gliclazid hat ein niedrigeres Hypoglykämierisiko im Vergleich zu den meisten anderen Sulfonylharnstoffen. Eine Gliclazid-basierte große randomisierte Outcome-Studie konnte eine signifikante Reduktion mikrovaskulärer Ereignisse zeigen (ADVANCE). In den letzten Jahren wurden Sulfonylharnstoffe in Zusammenhang mit einem erhöhten kardiovaskulären Risiko gebracht, welches in Metaanalysen für die angeführten Sulfonylharnstoffe nicht bestätigt werden konnte. Die Resultate der Metaanalysen konnten in der randomisiert, kontrollierten CAROLINA-Studie bestätigt werden, letztlich wurde bei der Verabreichung von Glimepirid kein Signal für ein erhöhtes kardiovaskuläres Risiko beobachtet.

### Glinide

Glinide (Repaglinid) führen zu einer gegenüber Sulfonylharnstoffderivaten kürzer dauernden prandialen Insulinsekretion mit einer größeren Flexibilität hinsichtlich der Nahrungsaufnahme. Analog zu den Sulfonylharnstoffen besteht jedoch auch ein Hypoglykämierisiko. Die HbA_1c_-Reduktion in der Monotherapie beträgt ca. 1,0 %. Diese Präparate sind unmittelbar präprandial einzunehmen. Positive, kardiovaskuläre Endpunktstudien liegen für die Glinide nicht vor.

Eine tabellarische Auflistung der einzelnen Medikamentenklassen finden Sie in Tab. 1.

### Insuline

Nach Ausschöpfung der nicht insulinbasierten, blutzuckersenkenden Therapieprinzipien stellt die Basalinsulintherapie eine einfache und gleichzeitig auch sichere Möglichkeit für den Einstieg in eine Insulintherapie dar. Kann unter dieser Therapie das individuell festgelegte Therapieziel nicht erreicht werden, so sollte je nach Wünschen und Bedürfnissen des Patienten eine Intensivierung der Therapie mithilfe eines zusätzlich verabreichten, prandialen Insulins oder mittels Mischinsulin erfolgen (siehe Leitlinie Insulintherapie, Tab. 1).

## Evidenzlage

Der epidemiologische Zusammenhang zwischen dem Ausmaß der Hyperglykämie und dem Auftreten mikro- und makrovaskulärer sowie zellulärer Komplikationen ist absolut gesichert.

Die zentrale Evidenz der UKPDS ist, dass eine intensivierte Therapie mit Insulin oder Sulfonylharnstoffen einer konventionellen Therapie mit primär Diät im Hinblick auf Komplikationen überlegen ist, wobei eine Verbesserung des HbA_1c_ um 0,9 % erreicht wurde [5].

Ein spezifischer Substanzvorteil stellte sich nur für Metformin als Therapie der 1. Wahl bei übergewichtigen Patienten dar [6]. In dieser Gruppe wurden Myokardinfarkte sowie Diabetes-assoziierte Mortalität und Gesamtmortalität signifikant gesenkt. Die Follow-up-Untersuchung der UKPDS-Population legt nahe, dass durch intensivierte Therapie langfristig die Gesamtmortalität gesenkt werden kann [6]. Ebenso legt diese Untersuchung die Existenz eines „metabolischen Gedächtnisses“ bei frisch manifestierten Patienten mit Typ 2 Diabetes nahe. Ähnliche Resultate wurden auch bei der EASD Konferenz (2022) für die 44 Jahre Follow Up Daten gezeigt.

UKPDS [6], ADVANCE [7], ACCORD [8, 9] legen zusammenfassend nahe, dass eine gute Blutzuckerkontrolle durch intensivierte Therapiestrategien, möglichst unmittelbar nach Diagnosestellung, erreicht und ohne schwere Hypoglykämien und exzessive Gewichtszunahme aufrechterhalten werden sollte. Langzeitbeobachtungen der Studien haben gezeigt, dass eine kardiovaskuläre Risikoreduktion von ca. 20 % erreicht werden kann [10]. In diesen Studien hatten die Patientengruppen mit den tiefsten HbA_1c_-Werten auch die niedrigste Mortalitätsrate pro Jahr.

### Kardiovaskuläre Endpunktstudien

Die EMPA-REG-Outcome-Studie [11] zeigte eine signifikante Reduktion des primären Endpunktes (kardiovaskulärer Tod, nicht-tödlicher Myokardinfarkt oder nicht-tödlicher Insult) durch Empagliflozin verglichen mit Placebo (HR 0,86; CI 0,74–0,99, *p* = 0,04). Kardiovaskulärer Tod (HR 0,62; CI 0,49–0,77, *p* < 0,001) und Gesamtmortalität (HR 0,68; 0,57–0,82; *p* < 0,001) wurden bei den, in der Studie eingeschlossenen Patienten mit kardiovaskulären Vorerkrankungen (KHK 75 %, Herzinfarkt 43 %, Schlaganfall 26 %, PAVK 23 %, Herzinsuffizienz 10 %) unter Empagliflozin signifikant im Vergleich zu Placebo gesenkt. Auch die Hospitalisierungsrate für Herzinsuffizienz sank unter Empagliflozin um ein Drittel (HR 065; 0,50–0,85; *p* = 0,002), das Auftreten von nicht-tödlichem Herzinfarkt, instabiler Angina pectoris und nicht-tödlichem Schlaganfall wurden hingegen nicht signifikant beeinflusst.

Die substanzspezifische, kardiovaskuläre Sicherheit von Dapagliflozin wurde in der DECLARE-TIMI-58-Studie an 17.160 Patienten untersucht, wobei 10.186 Patienten am Beginn der Studie keine kardiovaskuläre Erkrankung aufwiesen. Im Rahmen der primären Ergebnisanalyse konnte die kardiovaskuläre Sicherheit von Dapagliflozin bestätigt werden. Darüber hinaus wurde eine signifikante Reduktion kardiovaskulärer Todesfälle und Hospitalisationen aufgrund von Herzinsuffizienz registriert (HR 0,83; 0,73–0,95; *p* = 0,005). Dieser Effekt war hauptsächlich durch eine Reduktion der Hospitalisierung durch Herzinsuffizienz getrieben. Bemerkenswert ist, dass dieser Effekt auch in der Gruppe mit multiplen Risikofaktoren ohne vorhergehende kardiovaskuläre Erkrankung nachzuweisen war [12]. Dies unterstreicht die Relevanz von Dapagliflozin auch im Sinne eines Primärpräventionssettings.

Die Substanz Canagliflozin wurde mit Hilfe des CANVAS-Programmes untersucht. Mit Daten von 10.142 Patienten (2/3 der Patienten hatte eine bekannte koronare Herzkrankheit) konnte eine signifikante Reduktion des primären Endpunktes (HR 0,86; CI 0,75–0,97) gezeigt werden [13].

Im Rahmen der VERTIS CV Studie wurden kardiovaskuläre Effekte von Ertugliflozin bei 8246 Patienten mit Typ-2-Diabetes und atherosklerotischer kardiovaskulärer Vorerkrankung untersucht. Über einen Nachbeobachtungszeitraum von 3,5 Jahren konnte im primären Endpunkt (3-Punkt-Mace) die Nicht-Unterlegenheit im Vergleich zu Placebo signifikant dokumentiert werden (HR 0,97; 05 % CI: 0,85–1,11 *p* < 0,001). Der fehlende Nachweis einer Überlegenheit im Vergleich zu Placebo, insbesondere im kombinierten Endpunkt kardiovaskulärer Tod oder Hospitalisierung wegen Herzinsuffizienz ist doch recht überraschend, da man basierend auf den Daten der anderen SGLT-2-Hemmer eigentlich von einem Klasseneffekt ausgegangen wäre [14].

Hinsichtlich der GLP-1-Analoga, liegen für Liraglutid positive Daten aus einer kardiovaskulären Endpunktstudie vor. In der LEADER-Studie bewirkte Liraglutid eine signifikante Reduktion kardiovaskulärer Todesfälle und weiterführend eine Reduktion des präspezifizierten Endpunktes (HR 0,87; 0,78–0,97; *p* = 0,01 für superiority). Weiters konnte ein präspezifizierter, kombinierter renaler Endpunkt ebenso signifikant reduziert werden. Am Beginn der Studie hatten 81 % der Patient:innen eine bekannte kardiovaskuläre Erkrankung [15]. Nur bei diesen Patient:innen konnte eine kardiovaskuläre Risikoreduktion durch Liraglutid gezeigt werden.

Die „Researching Cardiovascular Events with a Weekly Incretin in Diabetes (REWIND)“ Studie, untersuchte den GLP-1-Rezeptoragonisten Dulaglutid. Diese Studie rekrutierte Patient:innen über ein breites kardiovaskuläres Risikospektrum, beginnend mit Menschen mit Typ 2 Diabetes und kardiovaskulären Risikofaktoren bis hin zu Personen mit Typ 2 Diabetes und manifester kardiovaskulärer Erkrankung. Interessant ist auch das Faktum, dass in der REWIND-Studie mit 9,5 % (< 81 mmol/mol) zwar eine obere HbA_1c_ Einschlussgrenze gesetzt wurde, jedoch keine untere. Das mediane HbA_1c_ der Gesamtstudienpopulation lag bei Studienbeginn bei 7,2 % (IQR 6,6–8,1 %).

In der im Median 5,4 Jahre dauernden Nachbeobachtungszeit konnte der primäre Endpunkt, nämlich der kombinierte Endpunkt aus kardiovaskulärem Tod, nicht-tödlichem Myokardinfarkt und nicht-tödlichem Schlaganfall (3-Punkt MACE) mittels Dulaglutid um relative 12 % reduziert werden (HR 0,88, 95 %-Konfidenzintervall 0,79–0,99). Es zeigte sich kein Unterschied in der Effektgröße zwischen jenen mit vorbestehender kardiovaskulärer Erkrankung und jenen ohne. Die Reduktion in der Gesamtmortalität erreichte keine statistische Signifikanz (HR 0,90 (0,80–1,01)). Die Reduktion des primären Endpunktes war unabhängig vom Ausgangs-HbA_1c_ in der Studie [16].

Für Semaglutid bestätigt die SUSTAIN-6-Studie ebenfalls positive kardiovaskuläre Effekte. Der primäre Endpunkt trat in der Semaglutid-Gruppe signifikant geringer auf (HR 0,74; 0,58–0,95 *p* < 0,001 für Non-Inferiority), wobei nicht tödliche Schlaganfälle in der Semaglutidgruppe signifikant reduziert wurden (HR 0,61; 0,38–0,99; *p* = 0,04) Entgegen den positiven kardiovaskulären Effekten kam es unter Semaglutid zu gehäuften Manifestationen der diabetischen Retinopathie (Glaskörperblutung, Erblindung, intravitreale Injektionen oder Photokoagulation) HR 1,76, 1,11–2,78, *p* = 0,02 [17]. Das Risiko für Augenkomplikationen wird durch die sehr rasche Blutzuckersenkung mit dieser Substanz erklärt und trat vor allem bei Menschen mit hohen HbA_1c_-Ausgangswerten auf. Die PIONEER-6-Studie zeigte die kardiovaskuläre Sicherheit von oralem Semaglutid bezüglich des primären Endpunktes von kardiovaskulärem Tod, nicht-tödlichem Myokardinfarkt und nicht-tödlichem Schlaganfall (3-Punkt MACE) in einem kardiovaskulärem Hochrisikokollektiv (85 % hatten eine vorbestehende kardiovaskuläre Erkrankung oder chronische Nierenerkrankung). Eine statistisch signifikante Reduktion des 3‑Punkt MACE wurde aber verfehlt (HR 0,79 (0,57–1,11)) [18].

Die Datenlage für Pioglitazon ist hinsichtlich einer möglichen kardiovaskulären Prävention ebenso positiv. Für Pioglitazon existiert mit PROACTIVE eine positive Endpunktstudie [19], die hinsichtlich des sekundären Endpunktes schwerer kardiovaskulärer Ereignisse insgesamt und besonders für die Subgruppen der Patienten mit vorangegangenem Myokardinfarkt [20] oder Schlaganfall [21] deutliche Vorteile zeigt. Dieser sekundäre Endpunkt war gleich wie der primäre Endpunkt in den rezenten Cardiovascular Outcome Trials. Der primäre Endpunkt der PROACTIVE Studie, der auch weniger harte kardiovaskuläre Ereignisse wie Amputationen einschloss wurde nicht signifikant reduziert. Eine Metaanalyse für Pioglitazon unterstützt die möglichen, kardiovaskulär präventiven Eigenschaften [22]. In der IRIS Studie, welche bei Patient:innen mit rezentem Insult und Insulinresistenz allerdings ohne manifestem Diabetes mellitus Typ 2 durchgeführt wurde, bewirkte eine Therapie mit Pioglitazon eine signifikante Reduktion des Auftretens von erneuten ischämischen Insulten und Myokardinfarkt (HR 0,76; 0,62–0,93; *p* = 0,007) [23].

Unter einer Basalinsulintherapie mit Glargin konnte in der ORIGIN Studie bei rezent an Diabetes mellitus Typ 2 erkrankten Patienten keine signifikante Reduktion kardiovaskulärer Endpunkte gezeigt werden. Interessanterweise waren im Vergleich zur Kontrollgruppe die Hypoglykämierate und die Gewichtszunahme zwar signifikant erhöht aber nur gering ausgeprägt [24]. Für Glargin und Degludec konnte die kardiovaskuläre Sicherheit gezeigt werden ([25]; siehe Tab. 2).

**Table 2:** 

Studienname, Substanz, primärer Endpunkt (CVOT)			Sekundärer Endpunkt^a^					
	Prim. Endpunkt	MACE	Gesamt Mortalität	CV-Mortalität	Myokardinfarkt	Insult	Hosp. wg. Herzinsuffizienz	Renale Endpunkte^b^
EMPA-REG-OUTCOME, Empagliflozin	↓(3-MACE)	↓	↓	↓	=	=	↓	↓
CANVAS, Canagliflozin	↓(3-MACE)	↓	=	=	=	=	↓	↓
DECLARE, Dapagliflozin	↓(kardiovaskulärer Tod und HHI)	=	=	=	=	=	↓	↓
VERTIS-CV, Ertugliflozin	= (3-MACE)	=	=	=	=	=	↓	**=**
ELIXA, Lixisenatid	= (4-MACE)	=	=	=	=	=	=	**n.b.**
EXCSEL, Exenatid	= (3-MACE)	=	↓	=	=	=	=	**n.b.**
LEADER, Liraglutid	↓(3-MACE)	↓	↓	↓	=	=	=	↓
SUSTAIN‑6, Semaglutid s.c.	↓(3-MACE)	↓	=	=	=	↓	=	↓
REWIND, Dulaglutid	↓(3-MACE)	↓	=	=	=	↓	=	↓
PIONEER‑6, Semaglutid oral	= (3-MACE)	=	↓	↓	=	=	=	**n.b.**
SAVOR TIMI, Saxagliptin	= (3-MACE)	=	=	=	=	=	**↑**	**=**
EXAMINE, Alogliptin	= (3-MACE)	=	=	=	=	=	=	**=**
TECOS, Sitagliptin	= (4-MACE)	=	=	=	=	=	=	**=**
CARMELINA, Linagliptin	= (3-MACE)	=	=	=	=	=	=	**=**
UKPDS, Metformin Studie; Follow Up	↓(komb. Endpunkt)^c^	n.b.	↓	=	↓	=	=	**=**
PROACTIVE, Pioglitazon	= (komb. Endpunkt)^d^	↓	=	=	↓	↓	**↑**	=
ORIGIN, Glargin	= (3-MACE)	=	=	=	=	=	=	=
DEVOTE, Degludec	= (3-MACE)	=	=	=	=	=	=	=

*CVOT* cardiovascular outcomes trial, *n.b.* nicht berichtet

^a^ Hypothesengenerierend

^b^ Wie in der Hauptpublikation definiert

^c^ Jegliche Diabetes-bezogene klinische Endpunkte, Diabetes-bezogener Tod, Gesamtmortaltität

^d^ Kombinierter Endpunkt aus Gesamtmortalität, nicht-tödlichem Herzinfarkt, (einschließlich stummer Infarkte), nicht-tödlichem Schlaganfall, akutes Koronarsyndrom, endovaskuläre oder chirurgische Intervention der Koronarien oder Beinarterien, Amputation über dem Knöchel

### Herzinsuffizienz

Sowohl Dapagliflozin, Empagliflozin, als auch Canagliflozin haben in den jeweiligen Endpunktstudien deutliche Reduktionen in der Hospitalisierung aufgrund einer Herzinsuffizienz gezeigt.

In der DAPA-HF-Studie wurden Personen mit vorbestehender Herzinsuffizienz mit reduzierter Auswurffraktion eingeschlossen (ejection fraction < 40 % und Symptome entsprechend New York Heart Association (NYHA) Klasse II–IV und erhöhten N‑terminal pro-B-type natriuretic peptide – Spiegel). Ein vorbestehender Diabetes mellitus Typ 2 war kein verpflichtendes Einschlusskriterium. In der Dapagliflozin-therapierten Gruppe zeigte sich eine signifikante Reduktion des primären Endpunktes (Verschlechterung der Herzinsuffizienz – dies war entweder eine Hospitalisierung oder dringliche Visite mit intravenösen Herzinsuffizienztherapie – oder kardiovaskulärer Tod) (HR 0,74; 95 % CI 0,65–0,85; *p* < 0,01). Dieser Effekt war unabhängig davon, ob ein Diabetes mellitus Typ 2 vorbestehend war, oder nicht [26]. Neben der DAPA-HF-Studie liegen auch die Resultate der EMPEROR-Reduced-Studie, welche mit Empagliflozin durchgeführt wurde, vor. Insgesamt wurden 3730 Patient:innen, die an einer manifesten Herzinsuffizienz NYHA II–IV erkrankt waren und eine linksventrikulären Ejektionsfraktion ≤ 40 % hatten untersucht. Im Rahmen dieser, randomisiert kontrollierten Studie wurde Empagliflozin 10 mg mit Placebo zusätzlich zur etablierten, leitliniengerechten Therapie untersucht. Die mediane Nachbeobachtungszeit lag bei 16 Monaten, die Prävalenz des Diabetes mellitus lag bei 49,8 %. Unabhängig vom Vorliegen eines Diabetes mellitus konnte der primäre Endpunkt (Hospitalisierungsrate aufgrund von Herzinsuffizienz oder kardiovaskulärer Tod) durch die Gabe von Empagliflozin signifikant gesenkt werden (HR 0,75; 95 % CI: 0,65–0,86; *p* < 0,001) [27]. Entgegen den Resultaten von DAPA-HF konnte in der EMPEROR-Studie keine signifikante Reduktion des kardiovaskulären Todes alleine dokumentiert werden. Im direkten Vergleich lag die Ereignisrate für den primären Endpunkt in der EMPEROR-Studie höher als in DAPA-HF, was sich letztlich auch durch die Tatsache erklären lässt, dass die Patient:innen in der EMPEROR-Studie durchwegs fortgeschrittenere Stadien der Herzinsuffizienz aufwiesen.

Die Emperor-Preserved Studie untersuchte 5988 Patient:innen, deren linksventrikuläre Auswurffraktion > 40 % war und stellt die erste Studie eines Gliflozins in diesem Kollektiv dar. Letztlich bewirkte Empagliflozin eine signifikante Reduktion des primären Endpunktes (kardiovaskulärer Tod oder Hospitalisation aufgrund von Herzinsuffizienz) um 21 % (HR 0,79, 95 % CI 0,69–0,90) [28].

Beinahe analog zur EMPEROR-Preserved-Studie zeigte sich für Dapagliflozin in der DELIVER-Studie ebenfalls ein positiver Effekt bei Patient:innen mit einer LVEF > 40 %. Über einen Nachbeobachtungszeitraum von 2,3 Jahren konnte der primäre Endpunkt (Verschlechterung der Herzinsuffizienz oder kardiovaskulärer Tod) signifikant reduziert werden (HR 0,82 CI 0,73–0,92; *p* < 0,001) [29].

Diese Daten ergänzen und verstärken die Empfehlung, dass bei vorbestehender Herzinsuffizienz ein SGLT2-Hemmer mit Evidenz zur Reduktion von Herzinsuffizienz (siehe Abb 1 und Tab. 3), unabhängig vom HbA_1c_ eingesetzt werden sollte.

**Table 3:** 

Studienname, Substanz, primärer Endpunkt (CVOT)			Sekundäre Endpunkte		
	Prim. Endpunkt	Gesamt-Mortalität	CV-Mortalität	Hosp. wg Herzinsuffizienz	Renale Endpunkte
DAPA-HF, Dapagliflozin	↓Kardiovaskulärer Tod, ungeplante Hospitalisierung oder ungeplanter, ambulanter Kontakt mit intravenöser Therapie	↓	↓	↓	=^a^
EMPEROR reduced, Empagliflozin	↓Kardiovaskulärer Tod, Hospitalisierung wegen Herzinsuffizienz	=	=	↓	↓^b^
EMPEROR preserved, Empagliflozin	↓Kardiovaskulärer Tod, Hospitalisierung wegen Herzinsuffizienz	=	=	↓	=
DELIVER Dapagliflozin	↓Kardiovaskulärer Tod, Hospitalisierung oder ambulanter Kontakt wegen Herzinsuffizienz	=	=	↓	

^a^ Kombinierter Endpunkt aus: > 50 % Reduktion der eGFR für zumindest 28 Tage, ESKD oder renaler Tod

^b^ Kombinierter Endpunkt aus: anhaltender Reduktion der eGFR um 40 %, anhaltende eGFR von < 15 ml/min/1,73 m^2^ bei einer Baseline eGFR ≥ 30 ml/min/1,73 m^2^ oder anhaltende < 10 ml/min/1,73 m^2^ bei einer Baseline eGFR < 30 ml/min/1,73 m^2^, chronische Dialyse oder renale Transplantation

### Nephropathie

Die „Canagliflozin and Renal Events in Diabetes with Established Nephropathy Clinical Evaluation (CREDENCE)“ Studie war die erste renale Studie eines SGLT-2-Hemmers, der den primären, kombinierten Endpunkt von Dialysepflichtigkeit, eGFR < 15 ml/min/1,73 m^2^, Verdopplung des Serumkreatinins, renaler oder kardiovaskulärer Tod untersuchte. Es wurden Personen mit Diabetes mellitus Typ 2 und chronischer Niereninsuffizienz auf maximal tolerierter Dosis eines ACE-Hemmers oder Angiotensin-Rezeptorblockers mit einer Harn Albumin/Kreatinin-Ratio von 300–5000 mg/g und einer eGFR von 30–90 ml/min/1,73 m^2^ untersucht. Canagliflozin reduzierte im Vergleich zu Placebo den primären Endpunkt um 30 % (HR 0,70; 95 % CI 0,59–0,82; *p* < 0,001) [30].

In der DAPA-CKD-Studie wurden 4304 Patient:innen mit chronischer Niereninsuffizienz (eGFR 25–75 ml/min/1,73 m^2^ und einer Albumin – Kreatinin – Ratio von 200–5000 mg/g untersucht. Die Prävalenz des Diabetes lag bei 67,5 %. Die Studie wurde nach einer medianen Beobachtungszeit von 2,4 Jahren aufgrund der positiven Effekte von Dapagliflozin gestoppt. Die Gabe von Dapagliflozin konnte den primären Endpunkt (Abnahme der eGFR um mindestens 50 %, terminale Niereninsuffizienz oder Tod aufgrund einer kardiovaskulären oder renalen Ursache) signifikant reduzieren (HR 0,61; 95 % CI: 0,51–0,72; *p* < 0,001). Das Vorliegen eines Diabetes mellitus hatte keinen signifikanten Einfluss auf die positiven Effekte von Dapagliflozin hinsichtlich der gewählten Endpunkte [31].

Die EMPA-Kidney Studie untersuchte 6609 Patient:innen mit chronischer Niereninsuffizienz (eGFR 20–45 ml/min/1,73 m^2^ oder 45–90 ml/min/1,73 m^2^ und einer Albumin – Kreatinin Ratio von mindestes 200 mg/g). Während der medianen Nachbeobachtungszeit von 2 Jahren wurde durch die Gabe von Empagliflozin der Endpunkt (Progression der Niereninsuffizienz oder kardiovaskulärer Tod) signifikant gesenkt (HR 0,72; 95 % CI: 0,6–0,82; *p* < 0,01) [32].

Diese Daten unterstützen die Empfehlung, dass bei chronischer Niereninsuffizienz, SGLT-2-Hemmer mit Evidenz für Reduktion der Progression der chronischen Niereninsuffizienz unabhängig von der aktuellen Blutzuckersituation eingesetzt werden sollen (siehe Abb. 1 und Tab. 4).

**Table 4:** 

Studienname, Substanz, primärer Endpunkt (CVOT)			Sekundäre Endpunkte	
	Prim. renaler Endpunkt	Gesamt-Mortalität	CV-Mortalität	Kardiovask. Tod oder Hosp. wegen Herzinsuffizienz
DAPA-CKD, Dapagliflozin	↓^a^	↓	**=**	↓
CREDENCE, Canagliflozin	↓^b^	=	=	↓
EMPA – KIDNEY, Empagliflozin	↓^c^	=	=	=

^a^ Kombinierter Endpunkt aus: Abfall der eGFR um zumindest 50 %, ESKD, renaler oder kardiovaskulärer Tod

^b^ Kombinierter Endpunkt aus: anhaltende Verdopplung des Serumkreatinins, ESKD, renaler oder kardiovaskulärer Tod

^c^ Kombinierter Endpunkt aus: Abfall der eGFR < 10 ml pro Minute pro 1,73 m^2^, Abfall der eGFR > 40 % vom Ausgangswert, renaler Tod

## Interessenkonflikt

H. Abrahamian hat von folgenden Unternehmen, die auch fördernde Mitglieder der ÖDG sind, Forschungsunterstützungen und/oder Honorare erhalten: Amgen, Boehringer Ingelheim, Novo Nordisk, sanofi-aventis, Servier. Zusätzlich gibt H. Abrahamian an, dass für sie persönlich kein Interessenkonflikt vorliegt. F. Aberer erhielt Honorare für Vorträge von AMGEN, Sanofi, Novo Nordisk, Astra Zeneca und Boehringer Ingelheim und Forschungsgelder von Sanofi. H. Brath hat von folgenden Unternehmen, die auch fördernde Mitglieder der ÖDG sind, Forschungsunterstützungen und/oder Honorare erhalten: Abbott Diabetes Care, Amgen, AstraZeneca, Boehringer Ingelheim, Eli Lilly, Medtronic, MedMedia, MSD, Novartis, Novo Nordisk, Sandoz, Sanofi, Servier. J. Brix erhielt Honorare für Advisory Boards und Vorträge von Astra Zeneca, Boehringer-Ingelheim, Eli Lilly, Novo Nordisk, sanofi. M. Clodi hat von folgenden Unternehmen, die auch fördernde Mitglieder der ÖDG sind, Forschungsunterstützungen und/oder Honorare erhalten: Abbott Diabetes Care, AstraZeneca, Boehringer Ingelheim, Daiichi-Sankyo, Dexcom, Eli Lilly, Medtronic, MSD, Novartis, Novo Nordisk, Sanofi. H. Drexel gibt an, dass ein Interessenskonflikt besteht. P. Fasching erhielt Vortragshonorare und Honorare für Consulting bzw. Fortbildungsförderung (Kongressunterstützung) von: Astra Zeneca, Boehringer Ingelheim, Eli Lilly, Novo Nordisk, Sanofi, Viatris. Teilnahme an Klinischen Studien: Boehringer Ingelheim, Eli Lilly, Novo Nordisk, Sanofi. B. Föger hat von folgenden Unternehmen, die teilweise auch fördernde Mitglieder der ÖDG sind, Forschungsunterstützungen und/oder Honorare erhalten: Amgen, Astra-Zeneca, Böhringer-Ingelheim, Janssen, Lilly, MSD, Novartis, Novo-Nordisk, Sanofi. C. Francesconi: Leitlinien, Adboard und VTs Böhringer und Novo Nordisk. E. Fröhlich-Reiterer hat von folgenden Firmen Vortragshonorare, Forschungsgelder und Honorare für Beratungstätigkeit erhalten: Medtronic, Novo Nordisk, Eli Lilly, Sanofi und Merck. J. Harreiter: Forschungsförderung und Vortragshonorare bzw. Fortbildungsförderung (Kongressunterstützung): Astra Zeneca, Novo Nordisk, Böhringer Ingelheim. S. Hofer hat Vortragshonorare und Honorare für Beratungstätigkeit von folgenden Firmen erhalten: Eli Lilly, Insulet, Dexcom, Ypsomed, Medtronic. F. Hoppichler hat von folgenden Unternehmen, die auch fördernde Mitglieder der ÖDG sind, Forschungsunterstützungen und/oder Honorare erhalten: Astra Zeneca, Boehringer Ingelheim, Eli Lilly, Novartis, Novo Nordisk, Sanofi Aventis. J. Huber hat von folgenden Unternehmen, die auch fördernde Mitglieder der ÖDG sind, Fortbildungsunterstützung und/oder Honorare für Vorträge und Beratung erhalten: Eli Lilly, Novartis, Novo Nordisk, Boehringer Ingelheim, Astra Zeneca, Sanofi-Aventis, Merck Sharp & Dohme, Daiichi-Sankyo, Viatris. Zusätzlich gibt J. Huber gibt an, dass kein Interessenkonflikt besteht. S. Kaser hat von folgenden Unternehmen, die z. T. auch fördernde Mitglieder der ÖDG sind, Forschungsunterstützungen und/oder Honorare erhalten: AstraZeneca, Boehringer Ingelheim, Eli Lilly, Merck Sharp & Dohme, Novartis, Novo Nordisk, Viatris. A. Kautzky-Willer hat von folgenden Unternehmen, die auch fördernde Mitglieder der ÖDG sind, Forschungsunterstützungen und/oder Honorare erhalten: AstraZeneca, Amgen, Boehringer Ingelheim, Eli Lilly, Merck Sharp & Dohme, Novartis, NovoNordisk, sanofi-aventis. M. Lechleitner: Vortragstätigkeit und Teilnahme an Advisory Boards: Boehringer Ingelheim, Daiichi Sankyo Austria, Sanofi, Novo Nordisk. B. Ludvik erhielt Honorare für Vorträge, die Teilnahme an Advisory Boards und Forschungsunterstützung von: Amgen, Astra Zeneca, Bayer, Boehringer Ingelheim, Daiichi Sankyo, Eli Lilly, MSD, Novartis, Novo Nordisk, Sanofi A. Luger: Keine Interessenskonflikte. J. K. Mader erhielt Vortragshonorare von: AstraZeneca, Abbott Diabetes Care, Böhringer-Ingelheim, Eli Lilly, Medtronic Minimed, Medtrust AG, Menarini, Merck Sharp & Dohme, Nintamed, Novo Nordisk, Roche Diabetes Care, Sanofi-Aventis, Servier, Viatris und Ypsomed. Advisory Board: Abbott Diabetes Care, Böhringer-Ingelheim, Eli Lilly, embecta, Medtronic Minimed, Novo Nordisk, Pharmasense, Roche Diabetes Care, Sanofi-Aventis, Viatris. Gesellschafterin decide Clinical Software GmbH. Forschungsgelder von A. Menarini Diagnostics, B. Braun, Metronom Health, Novo Nordisk, Profusa Inc., Roche Diabetes Care, Sanofi-Aventis. B. Paulweber hat von folgenden Unternehmen Forschungsunterstützungen und/oder Honorare erhalten: Amgen, AstraZeneca, Boehringer-Ingelheim, Eli Lilly, Merck Sharp Dohme, Mylan, Novartis, Novo Nordisk, Pfizer, Sanofi-Aventis. Th. Pieber: Direktor des Institutes für Biomedizin und Gesundheitswissenschaften (HEALTH) von JOANNEUM RESEARCH. Wissenschaftlicher Vorstand von CBmed (Zentrum für Biomarkerforschung) erhielt Vortragshonorare von: Novo Nordisk. Mitglied von Advisory Boards von: Arecor, Novo Nordisk, Sanofi, AstraZeneca, Adocia. R. Prager: klinische Studien Novo Nordisk. B. Rami-Merhar hat in den letzten 36 Monaten von folgenden Firmen Honorare/Forschungsgelder erhalten: Eli Lilly, Medtronic, Insulet, Abbott, Sanofi. G. Rega-Kaun legt offen, dass sie von folgenden Unternehmen, die teils auch fördernde Mitglieder der ÖDG sind, Honorare für Vorträge und/oder Beratertätigkeit bzw. Fortbildungsunterstützung erhalten hat: Amgen, Astra Zeneca, Boehringer Ingelheim, Daiichi Sankyo, Eli Lilly, MSD, Mylan, Novartis, Novo Nordisk, Sanofi Aventis. Zusätzlich gibt G. Rega-Kaun an, dass für sie persönlich kein Interessenkonflikt vorliegt. M. Resl hat von folgenden Unternehmen, die teils auch fördernde Mitglieder der ÖDG sind, Honorare für Vorträge und/oder Beratertätigkeit bzw. Fortbildungsunterstützung erhalten: Boehringer Ingelheim, Mylan, Dexcom, Abbott. C. Ress received speaker’s honoraria and congress support from AstraZeneca, Novo Nordisk, Pfizer, Boehringer Ingelheim, Amryt and Sanofi. M. Riedl: Eli Lilly, Boehringer Ingelheim, Novo Nordisk. M. Roden hat von folgenden Unternehmen Forschungsunterstützungen und/oder Honorare erhalten: Allergan, Astra Zeneca, Boehringer-Ingelheim, Bristol-Myers Squibb, Eli Lilly, Fishawack Group, Gilead, Genentech, Intercept Pharma, Inventiva, Merck, Novartis, Novo Nordisk, Nutricia/Danone, Pfizer, Poxel, Prosciento, sanofi-aventis, Target RWE. Ch. Saely: Astra Zeneca, Böhringer Ingelheim, Lilly, Merck, MSD, Mylan, Novartis, Sanofi-Aventis. Ch. Schelkshorn: Kongress Einladungen, Vorträge, sowie Adboards von Böhringer Ingelheim, Eli Lilly, Novo Nordisk, Sanofi, Astra Zeneca. G. Schernthaner: Keine Interessenskonflikte. H. Sourij hat von folgenden Unternehmen, die auch tlw. fördernde Mitglieder der ÖDG sind, Forschungsunterstützungen und/oder Honorare für Vorträge oder Beratertätigkeit erhalten: Amarin, Amgen, AstraZeneca, Boehringer-Ingelheim, Bayer, Daiichi Sankyo, Eli Lilly, Kapsch, Merck Sharp & Dohme, Novo Nordisk, Sanofi-Aventis. L. Stechemesser: Vortragshonorare und Honorare für Beratungstätigkeit von folgenden Firmen: Abbott, Amgen, Astra Zeneca, Boehringer Ingelheim, Dexcom, Eli Lilly, Medtronic, Medtrust, MSD, Mylan, Novartis, NovoNordisk, Roche Diabetes Care, Sanofi Aventis. Th. Stulnig hat von folgenden Unternehmen, die teils auch fördernde Mitglieder der ÖDG sind, Forschungsunterstützungen und/oder Honorare erhalten: Astra Zeneca, Boehringer-Ingelheim, Eli Lilly, Novo Nordisk, Sanofi, Takeda. H. Stingl legt offen, dass er von folgenden Unternehmen, die teils auch fördernde Mitglieder der ÖDG sind, Forschungsunterstützungen, Honorare für Vorträge und/oder Beratertätigkeit, bzw. Fortbildungsunterstützung im Rahmen der (dienst)rechtlichen Rahmenbedingungen (Einladung zu Kongressen) erhalten hat: Abbott, Amgen, AstraZeneka, Boehringer Ingelheim, Daiichi Sankyo, Eli Lilly, Menarini, MSD, Novo Nordisk, Novartis, Sanofi Aventis, Servier, Viatris. Zusätzlich gibt H. Stingl an, dass für ihn persönlich kein Interessenkonflikt vorliegt. H. Toplak hat Honorare für Vorträge und Beratung von Amgen, Böhringer-Ingelheim, Daiichi Sankyo, MSD, Novonordisk, Novartis, Pfizer, Sanofi und Viatris erhalten. Er hat darüber hinaus Forschungsunterstützungen von Amgen, Daiichi Sankyo, Novartis und Pfizer erhalten. T. C. Wascher hat von folgenden Unternehmen, die teils auch fördernde Mitglieder der ÖDG sind, Forschungsunterstützungen und/oder Honorare erhalten: Abbott, Amgen, Astra Zeneca, Boehringer-Ingelheim, Daiichi-Sankyo, Eli Lilly, MSD, Novartis, Novo Nordisk, Roche, Sanofi-Aventis. Y. Winhofer, R. Weitgasser und S. Zlamal-Fortunat geben an, dass kein Interessenkonflikt besteht.
